# Mortality Statistics, Chicago

**Published:** 1875-04

**Authors:** 


BOARD OF HEALTH, OFFICE SANITARY SUPT.
CHICAGO, March, 1875.

*Dr. W. Hay, Editor Journal:*

DEAR SIR—You will see by the following mortality report that the number of deaths have decreased one hundred and twenty-two compared with last month, and twenty-three compared with the corresponding month of last year. At present, we are comparatively free from small-pox, the few cases in the city are of a mild type ; we have had but three deaths from this cause since July last. At present a large number of cases of scarlatina and measles are present in the city, but as a rule are very light, but few deaths having occurred from them. I think we can congratulate ourselves on the healthy condition of the city—the rate for last month was only 11.22 per one thousand inhabitants.

Respectfully,

BEN. C. MILLER, *San. Supt.*

---

***Chicago Mortality Report for February, 1875.*** Reported by Dr. BEN. C. MILLER, Sanitary Superintendent.

| | | | |
|---|---|---|---|
| Accident, crushed | 4 | Apoplexy | 4 |
| " by fall | 1 | Asthma | 2 |
| " by railroad | 2 | Ascites | 1 |
| " by scalding | 1 | Atelectasis pulmonum | 1 |
| Abscesses | 1 | Bowels, intussusception of | 1 |
| " lumbar | 1 | Brain, congestion of | 6 |
| Anæmia | 4 | " inflammation of | 3 |
| Angina pectoris | 1 | Bronchitis | 10 |
| " gangrenous | 1 | " capillary | 9 |

| | |
|---|---|
| Cancer | 1 |
| “ of breast | 1 |
| “ of liver | 1 |
| “ of rectum | 1 |
| “ of stomach | 2 |
| “ of uterus | 1 |
| Consumption | 55 |
| Convulsions | 66 |
| Croup | 7 |
| “ membranous | 2 |
| Cyanosis | 2 |
| Cynanche trachealis | 1 |
| Debility, general | 4 |
| Delirium tremens | 1 |
| Diarrhœa | 2 |
| “ chronic | 1 |
| Diphtheria | 7 |
| Dropsy, general | 3 |
| “ pericardiac | 1 |
| Dysentery | 2 |
| Dyspepsia | 1 |
| Eczema | 1 |
| Endo-carditis | 1 |
| Enteritis | 11 |
| Entero-colitis | 1 |
| Exposure | 1 |
| Erysipelas | 5 |
| Exhaustion | 1 |
| Fever, puerperal | 8 |
| “ intermittent | 1 |
| “ remittent | 2 |
| “ scarlet | 7 |
| “ typhoid | 8 |
| Frost bites | 2 |
| Gangrene | 1 |
| Gastritis | 2 |
| Gastro-enteritis | 2 |
| Hæmorrhagica purpura | 1 |
| Heart, disease of | 1 |
| “ dropsy of | 2 |
| “ organic disease of | 2 |
| “ hypertrophy of | 1 |
| “ rheumatism of | 3 |
| Heart, valvular disease of | 4 |
| Hydrocephalus | 6 |
| Inanition | 8 |
| Injury at birth | 1 |
| Kidneys, disease of | 1 |
| “ Bright’s disease of | 2 |
| Laryngitis | 2 |
| Larynx, inflammation of | 1 |
| Liver, hypertrophy of | 1 |
| Lungs, congestion of | 10 |
| “ paralysis of | 1 |
| Malformation | 1 |
| Measles | 13 |
| Meningitis | 13 |
| “ cerebro-spinal | 5 |
| “ tubercular | 1 |
| Old age | 15 |
| Paralysis | 1 |
| Pericarditis | 1 |
| Peritonitis | 5 |
| “ puerperal | 1 |
| Pneumonia | 47 |
| “ typhoid | 1 |
| Pleurisy | 1 |
| Pyæmia | 2 |
| Rheumatism | 2 |
| Septicæmia | 1 |
| Small-Pox | 2 |
| Spina bifida | 1 |
| Spine, disease of | 1 |
| “ paralysis of | 1 |
| Suicide, shooting | 1 |
| “ cutting throat | 1 |
| Syphilis | 2 |
| Tabes mesenterica | 6 |
| Teething | 1 |
| Tetanus | 2 |
| Trismus | 1 |
| Tumor on neck | 1 |
| Toxæmia | 1 |
| Whooping cough | 2 |
| Total | 449 |

Premature births, 8 ; Still births, 66. Total, 74.

COMPARISON.

Deaths in February, 1875, 449 ; in January, 1875, 571. Decrease, 122. Deaths in February, 1874, 472. Decrease, 23.

AGES.

| | |
|---|---|
| Under one year | 155 |
| One year to two | 56 |
| Two years to three | 14 |
| Three “ “ four | 9 |
| Four “ “ five | 7 |
| Five “ “ ten | 20 |
| Ten “ “ twenty | 17 |
| Twenty “ “ thirty | 37 |
| Thirty years to forty | 37 |
| Forty “ “ fifty | 31 |
| Fifty “ “ sixty | 26 |
| Sixty “ “ seventy | 21 |
| Seventy “ “ eighty | 10 |
| Eighty “ “ ninety | 7 |
| Ninety “ “ one hundred | 1 |
| One hundred and upwards | 1 |
| Total | 449 |

| | | | | | |
|---|---|---|---|---|---|
| White | 438 | Males | 240 | Married | 133 |
| Colored | 11 | Females | 209 | Single | 316 |
| Total | 449 | Total | 449 | Total | 449 |

NATIVITIES.

| | | | | | |
|---|---|---|---|---|---|
| Belgium | 2 | England | 3 | Poland 1—Russia 1 | 2 |
| Bohemia | 5 | France | 1 | Scotland | 4 |
| Canada | 4 | Germany | 45 | Sweden | 7 |
| Native—Chicago | 66 | Holland | 3 | Switzerland | 2 |
| Foreign, " | 161 | Ireland | 39 | Wales | 1 |
| U. States, other parts | 87 | Italy | 1 | Unknown | 4 |
| Denmark | 2 | Norway | 10 | Total | 449 |

Deaths daily, 16. Mean thermometer, 15°. Rain fall, 1.84 inches.

MORTALITY BY WARDS.

| Wards. | No. Deaths. | Pop. in 1874. | Percentage. |
|---|---|---|---|
| 1 | 3 | 5,725 | one death in 1,908 |
| 2 | 6 | 4,830 | " " 805 |
| 3 | 19 | 14,861 | " " 782 |
| 4 | 12 | 15,361 | " " 1,280 |
| 5 | 25 | 20,078 | " " 803 |
| 6 | 52 | 35,916 | " " 691 |
| 7 | 31 | 31,722 | " " 1,023 |
| 8 | 35 | 29,143 | " " 832 |
| 9 | 26 | 31,654 | " " 1,217 |
| 10 | 6 | 17,385 | " " 2,897 |
| 11 | 11 | 14,022 | " " 1,275 |
| 12 | 14 | 16,792 | " " 1,199 |
| 13 | 14 | 17,892 | " " 1,278 |
| 14 | 15 | 16,720 | " " 1,115 |
| 15 | 43 | 45,545 | " " 1,059 |
| 16 | 25 | 21,922 | " " 877 |
| 17 | 23 | 20,777 | " " 903 |
| 18 | 26 | 21,392 | " " 823 |
| 19 | 6 | 4,677 | " " 779 |
| 20 | 4 | 8,995 | " " 2,249 |
| | 396 | Ratio of deaths to population in 1874, one death in 880 3-5. | |

| | | | |
|---|---|---|---|
| No. deaths in Wards | 396 | Mercy Hospital | 1 |
| Accidents | 8 | Protestant Orphan Asylum | 3 |
| County Hospital | 14 | St. Luke's Hospital | 3 |
| Foundlings' Home | 16 | Suicides | 2 |
| Hospital Alexian Bros. | 3 | | |
| Hahneman Hospital | 2 | Total | 449 |
| Home for Friendless | 1 | | |

